# 526 B-Frail: Using Frailty Screening to Improve Prognostication in Older Adults with Burn Trauma

**DOI:** 10.1093/jbcr/iraf019.155

**Published:** 2025-04-01

**Authors:** Joanna Lee, Sarah Hobgood, Michael Feldman, Monica Jinsi

**Affiliations:** Evans-Haynes Burn Center; Virginia Commonwealth University; Virginia Commonwealth University; Virginia Commonwealth University

## Abstract

**Introduction:**

Burn injuries significantly impact patients and families, leading to increased morbidity, reduced quality of life, and financial strain due to prolonged hospitalizations and surgeries. In specialized burn centers, older adults face in-hospital mortality rates between 8.7% to 24.4%. Existing prognostic models for burn patients consider factors such as age and total body surface area (TBSA) burned but often neglect frailty - a known predictor of outcomes in various conditions. The lack of frailty assessments in burn care highlights a critical gap in patient evaluation. This study aims to investigate the impact of frailty on clinical outcomes in geriatric burn patients, hypothesizing that integrating frailty and burn status will enhance prognostication of outcomes in geriatric burn patients.

**Methods:**

The existing FRAIL scale is effective for frailty assessment but lacks specificity for burn patients. To address this, we conducted a retrospective chart analysis of 77 geriatric burn patients using data from EPIC, including TBSA, inhalation injury, renal insufficiency, wound healing time, and readmission rates, alongside frailty indicators such as falls, functional dependence, and pre-existing conditions. Bivariate and multivariable regression analyses examined associations between these factors and outcomes: mortality, length of hospital stay (LOS), and discharge disposition.

**Results:**

Among the cohort, 44.47% were functionally dependent, 28.9% had inhalation injuries, and 34.7% had renal insufficiency. Regarding discharge disposition, 53.8% returned home, 30.8% required post-acute care, and 9.1% died in the hospital. Functional dependence significantly predicted discharge outcomes, with 40.6% of dependent patients returning home compared to 76.3% of independent patients (p = 0.002). Renal insufficiency also affected discharge, with only 28.6% of affected patients returning home compared to 72.9% without (p < 0.001). LOS was significantly influenced by TBSA (β = 1.476, p < 0.001) and renal insufficiency, with a mean LOS of 18.67 days for patients with renal insufficiency compared to 9.72 days for those without (p = 0.024). TBSA was the strongest predictor of in-hospital mortality, with each percentage increase associated with an 11.2% increase in the odds of death (p = 0.030).

**Conclusions:**

Our findings identify TBSA as a crucial predictor of in-hospital mortality, emphasizing its importance in clinical management. Functional dependence and renal insufficiency were key determinants of discharge disposition and LOS, emphasizing the need to also prioritize these factors in care plans.

**Applicability of Research to Practice:**

Despite limited sample size, this study highlights the need for incorporating frailty assessments into standard burn care. A refined B-FRAIL scale integrating these factors could improve predictions of mortality, LOS, and discharge planning, optimizing resource allocation and outcomes for geriatric burn patients.

**Funding for the Study:**

N/A

